# Modeling low-dose radiation-induced acute myeloid leukemia in male CBA/H mice

**DOI:** 10.1007/s00411-020-00880-9

**Published:** 2020-11-22

**Authors:** Sjors Stouten, Sjoerd Verduyn Lunel, Rosemary Finnon, Christophe Badie, Fieke Dekkers

**Affiliations:** 1 Netherlands National Institute for Public Health and the Environment, Bilthoven, The Netherlands; 2 Mathematical Institute, Utrecht University, Utrecht, 3508 TA The Netherlands; 3 Cancer Mechanisms and Biomarkers Group, Radiation Effects Department, Centre for Radiation, Chemical and Environmental Hazards, Public Health England, Didcot, OX11 ORQ UK

**Keywords:** Low-dose, Acute myeloid leukemia, CBA mice, Mathematical modeling, Ionizing radiation exposure, LDEF

## Abstract

The effect of low-dose ionizing radiation exposure on leukemia incidence remains poorly understood. Possible dose-response curves for various forms of leukemia are largely based on cohorts of atomic bomb survivors. Animal studies can contribute to an improved understanding of radiation-induced acute myeloid leukemia (rAML) in humans. In male CBA/H mice, incidence of rAML can be described by a two-hit model involving a radiation-induced deletion with Sfpi1 gene copy loss and a point mutation in the remaining Sfpi1 allele. In the present study (historical) mouse data were used and these processes were translated into a mathematical model to study photon-induced low-dose AML incidence in male CBA/H mice following acute exposure. Numerical model solutions for low-dose rAML incidence and diagnosis times could respectively be approximated with a model linear-quadratic in radiation dose and a normal cumulative distribution function. Interestingly, the low-dose incidence was found to be proportional to the modeled number of cells carrying the Sfpi1 deletion present per mouse following exposure. After making only model-derived high-dose rAML estimates available to extrapolate from, the linear-quadratic model could be used to approximate low-dose rAML incidence calculated with our mouse model. The accuracy in estimating low-dose rAML incidence when extrapolating from a linear model using a low-dose effectiveness factor was found to depend on whether a data transformation was used in the curve fitting procedure.

## Introduction

Many epidemiological studies have been conducted to elucidate the relationship between low-dose (LD) ionizing radiation (IR) exposure and leukemia incidence (Hsu et al. 2013; Preston et al. 1994; Pearce et al. 2012; Laurier et al. 2017). For radiological protection it is important to reliably quantify possible LD risks, to develop health and safety policies concerning IR exposure related to occupational hazards and public health. Data analyses of the Japanese atomic bomb survivors life-span study showed that both a linear-quadratic (LQ) and a preferred purely quadratic model can describe acute myeloid leukemia (AML) risk over a wide dose range (Preston et al. 1994; Richardson et al. 2009; Hsu et al. 2013). Different dose-response curves can often describe available high-dose (HD) data well but provide significantly different LD risk estimates after extrapolation.

The dose and dose-rate effectiveness factor (DDREF) has been introduced by the International Commission on Radiological Protection (ICRP) to account for possible overestimation of risk, when extrapolating from HD (rate) data to infer cancer risk possibly observed after LD (rate) exposure (ICRP 1991). The DDREF combines the concepts of the LD effectiveness factor (LDEF) and the dose-rate effectiveness factor (DREF). An examination of the LDEF to facilitate LD extrapolation will be considered here in the context of AML.

IR-induced AML (rAML) has been studied extensively in CBA/H mice due to very low background incidence and similarities with human AML (Major 1979; Verbiest et al. 2015). CBA/H mice have been exposed to acute X-ray doses between 0.25 and 6 Gy with maximum rAML incidence of about 22% following 3 Gy exposure (Major and Mole 1978; Major 1979; Mole et al. 1983). Occurrence of these rAML cases can largely be described by a two-hit model in which the gene Sfpi1 coding for the hematopoietic transcription factor PU.1 undergoes two mutations. Hematopoietic target cells turn pre-leukemic after acquiring an IR-induced hemizygous interstitial deletion on chromosome 2 (del2) with Sfpi1 copy loss (Bouffler et al. 1996, 1997; Silver et al. 1999). In time, these cells become malignant after accumulating a point mutation in the remaining Sfpi1 allele at codon R235 in the DNA-binding domain of PU.1 (Suraweera et al. 2005; Cook et al. 2004), resulting in clonal expansion and rAML onset (Bouffler et al. 1996; Verbiest et al. 2018b).

The target cell responsible for leukemogenesis still remains unidentified, but multiple cases have been made for hematopoietic stem and progenitor cells (HSPCs) (Hope et al. 2004; Taussig et al. 2005; Shlush et al. 2014; Passegué et al. 2003; Hirouchi et al. 2011; Verbiest et al. 2018b). Dekkers et al. (2011) developed a mathematical model capable of quantifying rAML incidence in CBA/H mice in which hematopoietic stem cells (HSCs) were assumed to be the target cells responsible for rAML development. This study was the first endeavor to model murine rAML in terms of the two-hit model of leukemogenesis. However, due to lack of data, some key features such as cell/animal survival and formation of del2 cells could not be incorporated, nor was the model applied to study LD rAML incidence. New data have since become available that allowed, in the present study, to include these aspects in the model.

In the present paper a new model for X/gamma-ray-induced AML following acute exposure in male CBA/H mice is presented. This model expands on previous modeling work from Dekkers et al. (2011) by using historical data to include dose-dependent death of mice from non-rAML causes and by defining cell survival and del2 formation in terms of the LQ model. It is shown that numerical solutions can be approximated with simple expressions to describe various aspects of dose- and time-dependent rAML onset. Using HD rAML model estimates in a fitting procedure, it was possible to quantify how accurate various functions describe LD incidence.

## Materials and methods

### Brief model description

**Fig. 1:**
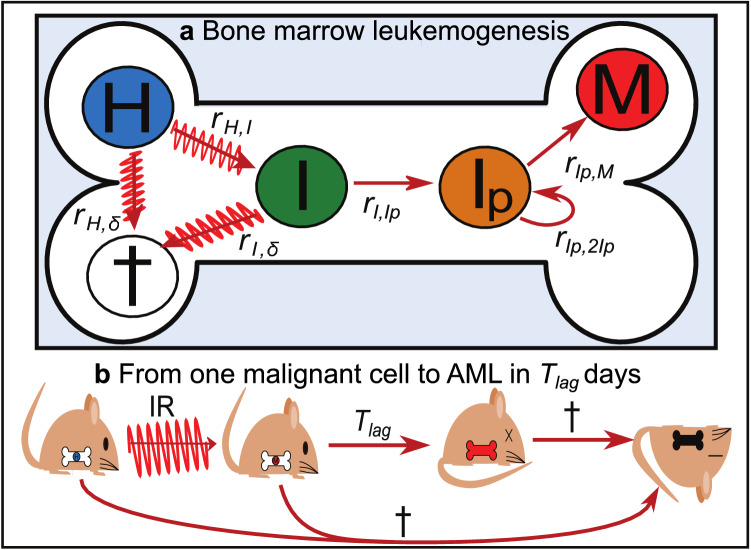
Overview of the rAML model. **a** Healthy bone marrow target cells *H* transform into pre-leukemic del2 intermediate cells *I* due to radiation-induced Sfpi1 copy loss. In time, intermediate cells *I* are selected from the stem cell pool to become proliferating del2 cells $I_p$, capable of forming malignant cells *M* after occurrence of the Sfpi1 point mutation. Cells *H* and *I* can additionally die as a consequence of radiation exposure. Transition rates *r* determine the pace at which cells progress through the model in response to radiation exposure. **b** Following total-body exposure mice may develop a malignant leukemic cell *M* that will result in rAML onset in $T_{lag}$ days, or they may die from other causes

Figure 1 shows an overview of the stochastic model used here to calculate dose-dependent rAML incidence in total body photon-irradiated male CBA/H mice in which HSCs are assumed to be responsible for leukemogenesis. A model for bone marrow leukemogenesis (Fig. 1a) is coupled to a model in which mice can die from either rAML or other causes (Fig. 1b). In the bone marrow, IR exposure causes healthy target cells *H* to either survive, die or transform into intermediate cells *I* carrying del2. These intermediate cells can give rise to actively proliferating del2 cells $I_p$ responsible for bringing about a malignant cell *M* after accumulation of the R235 point mutation in the remaining Sfpi1 allele. In *in silico* mice (Fig. 1b), clonal expansion starts after the first malignant cells has been formed, leading to rAML onset and diagnosis over the course of $T_{lag}$ days. Note that it is possible for mice to die from other causes before developing rAML.

**Fig. 2:**
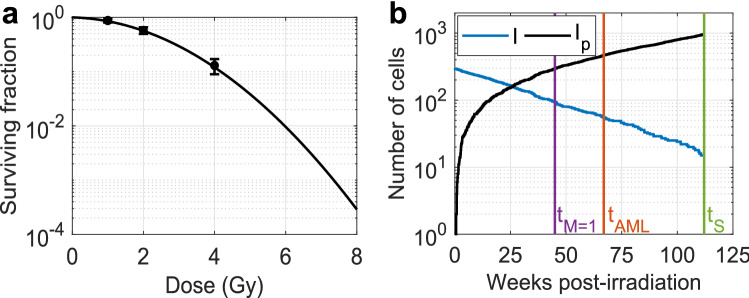
Clonal survival and bone marrow leukemogenesis. **a** HSC target cell survival is described through the linear-quadratic model. Data adapted from Mohrin et al. (2010) is shown with mean ± standard deviation $(n=3)$ of gamma-ray irradiated SLAM-HSC derived from C57BL/6 mice. **b** Following 3 Gy exposure, surviving bone marrow intermediate cells *I* carrying del2 (blue) are selected to enter proliferating compartment $I_p$ (black). The vertical purple line indicates the time at which the first malignant cell *M* is formed from $I_p$ due to the occurrence of the R235 point mutation, this *in silico* mouse will be diagnosed with rAML $T_{lag}$ days later (vertical red line). The vertical green line depicts the time at which this mouse would have died from another cause

**Fig. 3:**
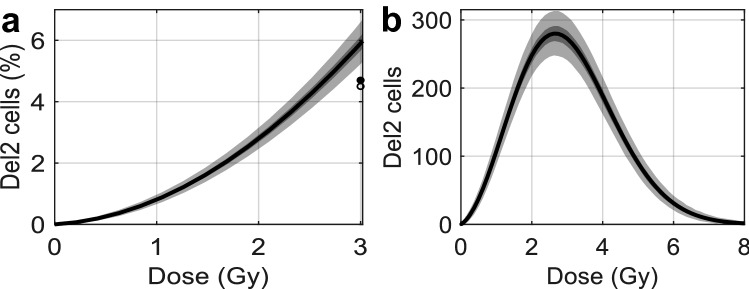
Formation of radiation-induced intermediate del2 cells present directly after exposure. **a** Mean percentage of del2 target cells is shown by the black curve and shaded areas represent 50% (dark shades) and 95% (light shades) of all model predictions. Data points represent *in vivo* (open circle) and *in vitro* (filled circle) measurements of relative del2 formation among X-ray irradiated LSK cells (Olme et al. 2013a). **b** The dose-response curve for the number of radiation-induced del2 cells reaches a maximum following 2.6 Gy exposure. Further increasing the dose results in more cell death, hence explaining the decrease in del2 cell formation

**Fig. 4:**
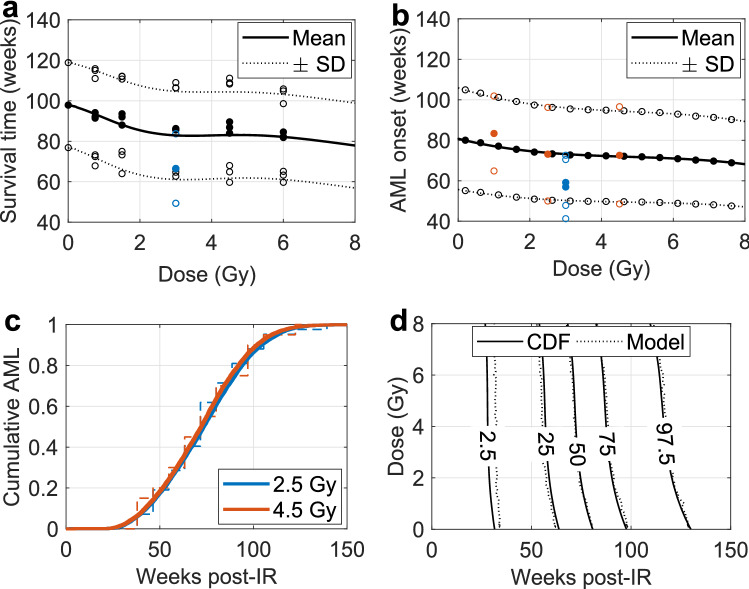
Dose- and time-dependent rAML onset. **a** Mean mouse survival time ± standard deviation (SD) is shown to decrease as a function of dose for model predictions (mean: solid curve; mean ± SD: dotted curve) and experimental observations (mean: filled circles; mean ± SD: open circles). Mean ± SD (black circles) are based on 40 to 61 CBA/H mice per dose and over 800 mice for the control (Major 1979), blue circles represent measurements from Olme et al. (2013b). **b** Model predictions (black) for the dose-dependent decrease in mean time to rAML onset (filled circles) ± SD (open circles) are shown with third-order polynomial fits (mean: solid curve; mean ± SD: dotted curve). Blue and red data points respectively correspond with the time of rAML onset from experiments conducted by Olme et al. (2013b) and Mole et al. (1983). **c** Cumulative time-dependent rAML incidence following 2.5 (blue) and 4.5 (red) Gy exposure is in line with data from CBA/H mice irradiated with 2–3 Gy (dashed blue stairs) or 4.5 Gy (dashed red stairs) X-rays (Mole et al. 1983). **d** Percentiles for cumulative rAML incidence can be described in a dose- and time-dependent manner through a normal cumulative distribution function (CDF, solid) used to approximate the model solutions (dotted)

**Fig. 5:**
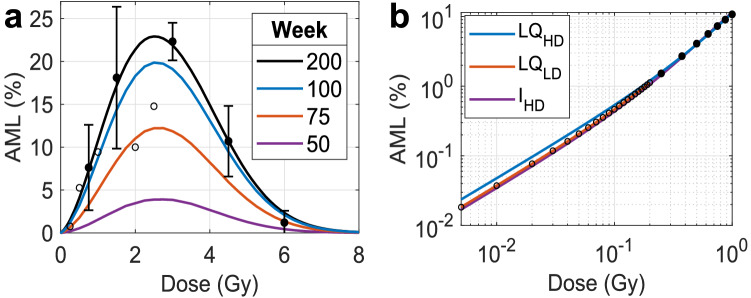
The rAML dose-response curve for X-ray irradiated CBA/H mice. **a** The modeled high-dose rAML incidence curve (black) describes the available data quite well: filled circles represent mean± standard deviation of 4 experiments conducted by Major (1979) and Mole et al. (1983); open circles represent data points from Mole et al. (1983) not included in the fitting procedure. Cumulative rAML percentage calculated in week 50, 75 and 100 are respectively depicted by the purple, red and blue curves. **b** The low-dose rAML estimates calculated with the model (open circles) increased linear-quadratically (LQ) with dose (red). Low-dose rAML model predictions can be estimated quite well by the LQ model (blue) and in terms of del2 formation *I* (purple) when only high-dose rAML estimates (black-filled circles) were made available in the fitting procedure to subsequently extrapolate from

Intercompartmental transition rates *r* (number of cells per hour) are the backbone of the model and are used in a stochastic tau-leap algorithm developed by Cao et al. (2006). During each time leap step $\tau$, the algorithm determines how many cells *H*, *I* and $I_p$ move along each of the arrows into a new compartment. The tau-leap algorithm was developed as a fast alternative to the relatively slow Gillespie stochastic simulation algorithm, which can generate sample trajectories distributed in accordance with the solution of the master equation (Gillespie 1976, 1977, 2001). Thus, by running the model once, one simulates stochastic continuous-time discrete-state trajectories for cells *H*, *I* and $I_p$ corresponding to the bone marrow response of a single irradiated *in silico* mouse.

### Modeling cell survival

IR-induced loss of clonal potential is based on the classical LQ model for describing clonogenic survival (Kellerer and Rossi 1974; Chadwick and Leenhouts 1973):

1 $$\begin{aligned} S(L(D))=e^{-L(D)}=e^{-(\alpha D + \beta D^2)}, \end{aligned}$$

where *L*(*D*) can be considered as the average number of IR-induced lethal lesions present per cell after receiving dose *D* (Gy). Cells are exposed with a constant dose-rate $\dot{D}$ (Gy/h) from $t=0$ to $t=D/\dot{D}=T$ hours. The lethal lesion formation rate follows from:

2 $$\begin{aligned} \dot{L}(t) = {\left\{ \begin{array}{ll} \alpha \dot{D} + 2 \beta \dot{D}^2t &{} \text {if } 0\le t \le T\\ 0 &{} \text {otherwise}. \end{array}\right. } \end{aligned}$$

Note that any lesions formed in the absence of radiation were disregarded. The rate at which healthy cells lose clonal potential, $r_{H,\delta }$, can be described in terms of the lethal lesion formation rate (Zaider and Minerbo 2000):

3 $$\begin{aligned} r_{H,\delta }(t) = \dot{L}(t)H(t) . \end{aligned}$$

The same rate for intermediate cells $r_{I,\delta }(t)$ was acquired by substituting *I*(*t*) for *H*(*t*).

### Del2 formation rate

The del2 interstitial deletion with Sfpi1 copy loss responsible for the transition from healthy cells to intermediate cells is a chromosomal aberration. The total number of chromosome aberrations can be described in terms of the aforementioned number of lethal lesions *L* because they are linearly correlated (McMahon 2018). Furthermore, the number of interstitial deletions present following radiation exposure can be described in terms of the number of chromosome aberrations (Cornforth et al. 2002). Based on these observations it is assumed that del2 constitute a fixed, dose-independent, fraction of the total number of chromosomal aberrations formed. The rate at which healthy cells *H* transform into intermediate cells *I* is then proportional to the rate at which healthy cells die (Eq. (3)):

4 $$\begin{aligned} r_{H,I}(t) = \mu_{del2} \dot{L}(t)H(t) \end{aligned}$$

The parameter $\mu _{del2}$ is a dimensionless scalar relating IR-induced formation of chromosome aberrations to the del2-induction rate. By using this rate it is assumed that all del2 cells are formed during exposure.

Since only acute exposure is considered here it is possible to derive an expression for the initial number of intermediate cells *I* at time $t=T\approx 0$ by using rates $r_{H,\delta }$, $r_{H,I}$ and $r_{I,\delta }$. Doing so reduces computation time because one only needs to track cells *I* and $I_p$ in time until the mouse dies or until the first malignant cell *M* is formed. The initial condition for cell population *I* can be found by solving the following ordinary differential equations for cells *H* and *I*:

5 $$\begin{aligned} \dot{H}&=-r_{H,\delta }-r_{H,I} \end{aligned}$$

6 $$\begin{aligned} \dot{I}&=-r_{I,\delta } +r_{H,I}. \end{aligned}$$

Note that the transitioning from *I* to $I_p$ can be ignored because any dose of interest is absorbed almost instantaneously. Solving these equations with initial conditions $H(0)=H_0$ and $I(0)=0$ yields the following expressions at time $t=T\approx 0$:

7 $$\begin{aligned} H(D)&= H_0 e^{- (\alpha D + \beta D^2 )(1+ \mu _{del2} )} \end{aligned}$$

8 $$\begin{aligned} I(D)&= H(D) \big (e^{\mu _{del2}(\alpha D + \beta D^2 )} -1 \big ), \end{aligned}$$

where $H_0\approx 15670$ HSCs (Staber et al. 2013). It is assumed that *I*(*D*) represents the mean of a Poisson distribution reflecting the number of del2 cells present after *T* hours of exposure, thus yielding the initial condition: $I(0)\sim$Pois(*I*(*D*)).

### Proliferation of pre-leukemic cells

Proliferation of the pre-leukemic subpopulation *I* is assumed to be negligible in the initial stage after IR exposure. This assumption is supported by two observations. Firstly, transplantation of irradiated Lin^-^c-Kit^+^Sca-1^+^ (LSK) cell populations containing del2 cells into host CBA/H mice leads to reduced repopulation of host animals compared to non-irradiated cells four months after exposure. This indicates that del2 cells do not have a proliferative advantage in the early stages following IR exposure (Olme et al. 2013a). Secondly, HSCs have very low proliferation rates (Manesso et al. 2013).

It has been proposed that approximately 9 months post-IR exposure, pre-leukemic cells with chromosome 2 abnormalities have an increased probability of being selected from the stem cell pool into the proliferating compartment (Bouffler et al. 1997). It is thus assumed here that non-proliferating intermediate cells *I* are selected from the stem cell pool into the proliferating intermediate cell compartment $I_p$ with a time-constant of $T_{pool}=9$ months. The corresponding transition rate then follows from:

9 $$\begin{aligned} r_{I,I_p}(t) = \frac{I(t)}{T_{pool}}. \end{aligned}$$

Thus assuming that, after 9 months on average, about 63% of the intermediate cells *I* moved into the proliferating intermediate cell compartment $I_p$. Note, however, that this rate is a simplification of a possible 9-month delay. Once selected from the stem cell pool, the proliferating pre-leukemic population $I_p$ is assumed to exhibit exponential growth through:

10 $$\begin{aligned} r_{I_p,2I_p}(t) = bI_p(t). \end{aligned}$$

Similarly to Dekkers et al. (2011), it is assumed that the proliferation rate is independent of dose. This is, in the early stages (at least 4 months) following exposure, supported by the aforementioned observation that transplantation of irradiated LSKs into host mice does not result in a growth advantage compared to controls (Olme et al. 2013a). Note, however, that the growth rate does *implicitly* depend on dose: the more radiation-induced del2 cells are formed, the larger the pool of proliferating pre-leukemic cells will become.

### Malignant cell formation and diagnosing rAML

The R235 point mutation responsible for malignant cell transformation has mainly been found in del2 hematopoietic cells and occurs late in the leukemogenic process (Verbiest et al. 2018b; O’Brien et al. 2020). Staber et al. (2013) showed that mice with heterozygous knockout of PU.1 had a $\sim$60% reduction in PU.1 mRNA levels compared to controls. Gault et al. (2019) suggested that an increased point mutation rate might be the result of (excessive) proliferation due to HSC cell cycle dysregulation following PU.1 loss (del2). It was therefore assumed that only proliferating intermediate cells $I_p$ can transform into malignant cells *M* through the rate:

11 $$\begin{aligned} r_{I_p,M}(t) = \mu _{ Sfpi1 } I_p(t), \end{aligned}$$

where $\mu _{Sfpi1}$ is the Sfpi1 point mutation rate. It should be noted that alternative explanations such as IR-induced genomic instability and/or oxidative stress might be responsible for the Sfpi1 point mutation (Wright 1998; Ishikawa and Morisaki 2019). Both explanations can in principle also be modeled through the above mutation rate.

Once the first malignant cell *M* has been formed at time $t_{M=1}$, it is assumed that rAML onset occurs over the course of $T_{lag}$ days with diagnosis taking place at time $t_{AML} = t_{M=1}+T_{lag}$. However, this only occurs if diagnosis takes place because the survival time of the mouse, $t_s$, is larger than or equal to the time of rAML diagnosis, that is, $t_{AML}\le t_s$. $T_{lag}$ was assumed to be 22 weeks based on the observation that C57BL mice developed rAML with a median latency of about 22 weeks following conditional knockout of both PU.1 alleles (Metcalf et al. 2006), this is similar to the latency of 21.86 weeks found by Dekkers et al. (2011).

### Dose-dependent survival of mice

To avoid overestimation of rAML incidence one needs to consider death due to other causes as well. Dose-dependent death unrelated to rAML in CBA/H mice was simulated by sampling nonnegative survival times ($t_S$) in days from a skew normal distribution with parameters $\xi =786.43-17.45D$, $\omega =178.60$ and $\alpha =-1.013$.

Distribution parameters were set in accordance with the observation that survival time in unexposed male CBA/H mice follows a left-skewed distribution with a skewness of $-0.141$, a mean of 685 days and a standard deviation (SD) of 147 days. Skew normal distribution parameter $\xi$ is assumed to decrease linearly with dose to satisfy the observation that mean survival time decreases to approximately 580 days after 6 Gy X-ray exposure (Major 1979).

### Model implementation, data and fitting procedure

The adaptive tau-leap algorithm developed by Cao et al. (2006) was used to run the model in R version 3.5.0 (R Core Team 2018) using the *AdaptiveTau* package with an error control parameter of $\epsilon =0.01$. AML incidence was calculated using 100 and 10 million *in silico* mice per dose following LD ($D\le$ 0.2 Gy) and HD exposure respectively. All model results were plotted in MATLAB R2018a.

Fit parameters $\alpha$, $\beta$, $\mu _{del2}$, *b* and $\mu _ {Sfpi1}$ were determined by minimizing a residual sum of squares cost function using data on the mean AML onset time in weeks after 4.5 Gy exposure ($\tau$) and rAML incidence measurements (***A***) following 0.75, 1.5, 3, 4.5 and 6 Gy X-ray exposure in CBA/H mice (Major 1979; Mole et al. 1983). Incidence percentages were weighted ($\mathbf {w}$) by multiplying each residual with the corresponding fraction of mice used per measurement. The residual sum of squares cost function for a vector of fit parameters $\mathbf {p}$ is given by:

12 $$\begin{aligned} C(\mathbf {p})=&\sum _{i=1}^{n} w(i) \bigg (A(i)-\hat{A}(i,\mathbf {p}) \bigg )^2 + \nonumber \\&\bigg (\frac{\sum _{i=1}^{n} w(i)A(i)}{n\tau } \Big (\tau -{\hat{\tau }}(\mathbf {p})\Big ) \bigg )^2, \end{aligned}$$

where $\hat{A}(i,\mathbf {p})$ is the modeled percentage of rAML incidence corresponding to data point *i* out of the $n=20$ data points for parameters $\mathbf {p}$, and $\hat{\tau }(\mathbf {p})$ is the mean rAML onset time model estimate in weeks following 4.5 Gy exposure. Note that the residual $\tau -\hat{\tau }(\mathbf {p})$ of the rAML onset time was scaled by the average weighted percentage of rAML incidence and divided by $\tau$ to correct for the dimensions and thus include both quantities in the fitting procedure.

**Table 1:** Best-fit rAML model parameters reported with a bias-corrected 95% bootstrap confidence interval and the starting values used in the simulated annealing fitting procedure

Constant	Units	Start	Best-fit values
$\alpha$	Gy^-1^	0.01	0.0402 (0.0068; 0.181)
$\beta$	Gy^-2^	0.01	0.122 (0.0779; 0.163)
$\mu _ {del2}$	-	0.01	0.0498 (0.013; 0.388)
$\mu _ {Sfpi1}$	day^-1^	24$\cdot$10^-7^	2.26$\cdot$10^-6^ (3.91$\cdot$10^-7^; 7.04$\cdot$10^-6^)
*b*	day^-1^	24$\cdot$10^-4^	2.05$\cdot$10^-3^ (9$\cdot$10^-4^; 6.35$\cdot$10^-3^)

**Table 2:** Third-order polynomial fit parameters for the dose-dependent normal cumulative distribution function of the mean ($\mu _{t_{AML}}$) and standard deviation ($\sigma _{t_{AML}}$) of rAML onset times in weeks

Function	$c_0$	$c_1$	$c_2$	$c_3$
$\mu _{ t_{AML}}(D)$	80.8	$-$4.44	0.801	$-$0.0553
$\sigma _{t_{AML}}(D)$	25.1	$-$1.23	0.206	$-$0.0143

The parameter space was initially explored by minimizing the cost function through a simulated annealing algorithm with 10,000 iterations (R package: *GenSA*), using 1000 *in silico* mice per data point and initial educated guess values listed in Table 1. The optimal solution found with simulated annealing was then fed into the Nelder-Mead method for local optimization (R package: *dfoptim*) using 100,000 *in silico* mice per data point. Given the best-fit parameters and residuals, parameter uncertainty was determined by following a non-parametric residual-based bootstrap method proposed by Dogan (2007), in which 5000 Nelder-Mead fitting procedures (1000 *in silico* mice per data point) were performed on simulated data sets generated from the best-fit model output and the sampled residuals. Table 1 contains the bias-corrected 95% percentile bootstrap confidence intervals (Efron and Tibshirani 1986; Dogan 2007) calculated from the bootstrap samples. The number of *in silico* mice used per data point in a given fitting procedure was based on a balance between accuracy and computation time.

### Approximating model solutions and the LDEF

Numerical model solutions were approximated with simple analytical expressions to study properties of dose- and time-dependent rAML onset. The LD response curve for rAML incidence was approximated using an LQ model as well as a dose-dependent function in which incidence was taken to be proportional to the average number of del2 cells formed in a single mouse following exposure (Eq. 8). Note that an LQ model for the LD response-curve ($\alpha D + \beta D^2$) should not be confused with the LQ model for cell survival (Eq. 1). It was further assessed how well an LQ model can be used to infer model-derived LD rAML incidence ($D\le 0.20$ Gy) after fitting its parameters to model-derived HD rAML incidence estimates between 0.25–1.00 Gy. A linear model ($\alpha _L D$) was additionally fitted to the same HD rAML estimates to evaluate how well an LDEF improves LD extrapolations made with a linear model. The LDEF is calculated in dose $D_x=1$ Gy through: $LDEF=1+(\beta /\alpha ) D_x$ (Rühm et al. 2016), where $\alpha$ and $\beta$ are the LQ model parameters.

The numerical solution for time of rAML diagnosis was estimated using a normal cumulative distribution function (CDF) in which the mean and SD were modeled with third-order polynomials ($\sum _{k=0}^{3}c_k D^k$). A third-order polynomial was chosen because it contains the minimum number of parameters required to sufficiently describe the model solutions.

The parameters for the aforementioned functions were determined through a nonlinear least-squares method using the *nls* function of the *stats* package in R. For LD rAML incidence, the curves were fitted with(out) log transforming incidence estimates to study the effect of transformation on the predictive value. The residual sum of squares of log transformed rAML incidence values ($RSS_{log}$) was used as a measure for LD fit quality.

## Results

### Response of bone marrow cells to IR exposure

Loss of clonal potential of HSC target cells was described through the LQ model (Fig. 2a), yielding model predictions that are in line with gamma-irradiated Slam-HSC (LSK, Flk2^-^, CD150^+^, CD48^-^) survival data (Mohrin et al. 2010) not included in the fitting procedure. Figure 2b shows the simulated response of bone marrow cell populations in a single *in silico* mouse following 3 Gy exposure. Intermediate cells *I* (blue) are formed due to IR-induced del2-mediated Sfpi1 copy loss. Over time, intermediate cells *I* are selected from the stem cell pool into the proliferating compartment $I_p$ (black), resulting in malignant cell formation at time $t_{M=1}$ (vertical purple line) due to occurrence of the R235 point mutation. Time of diagnosis was found by registering the time at which the first malignant cell came into existence and adding time lag $T_{lag}$ (vertical red line). Diagnosis only took place if a mouse did not die from other causes during the time lag ($t_S$, vertical green line), that is, $t_{AML} = t_{m=1} + T_{lag} \le t_s$.

### Induction of del2

One of the two key steps required to explain murine rAML is the interstitial deletion on chromosome 2 with Sfpi1 copy loss. Dose-dependent del2 induction following IR exposure was modeled by deriving estimates for the mean Poisson distributed number of healthy (Eq. 7) and intermediate (Eq. 8) target cells present per mouse. Figure 3a shows a nonlinear dose-dependent increase in the mean percentage of del2 target cells (black), dark and light shaded regions respectively contain 50% and 95% of all model predictions. Shown CBA/H mouse data represents background corrected X-ray-induced relative del2 formation among LSK cells, which are comprised of about 10% HSCs, following *in vitro* (filled circle) and *in vivo* (open circle) exposure (Olme et al. 2013a). To compare these data points with the presented model solution it is assumed that del2 formation in LSK cells is similar to that in HSCs. The mean number of viable IR-induced del2 cells increases rapidly with dose until a maximum of about 280 cells are formed following 2.6 Gy exposure (Fig. 3b). Further increasing the dose results in enhanced cell killing, thus driving the total number of del2 cells towards zero.

### Dose- and time-dependent rAML onset

The survival time post-IR exposure was found to decrease in a dose-dependent manner, as shown by the drop in mean survival time (solid curve) from 98 to 82 weeks when the dose was increased from 0 to 6 Gy (Fig. 4a). The modeled mean survival time ± SD (dotted) is similar to experimental observations of X-ray irradiated male CBA/H mice (filled circles: mean; open circles: mean ± SD) from Major (1979), with a slight underestimation following 3 and 4.5 Gy exposure. The blue data points represent more recent survival time measurements of 3 Gy X-ray irradiated male CBA/H mice (Olme et al. 2013b).

A similar pattern was observed for the mean (filled circles) ± SD (open circles) time of rAML diagnosis, which decreased from 81 to 71 weeks when the dose was increased from 0 to 6 Gy (Fig. 4b). The model predictions (filled/open black circles) could be adequately described with dose-dependent third-order polynomials for the mean time of rAML onset ($\mu _{t_{AML}}(D)$, solid) ± SD ($\sigma _{t_{AML}}(D)$, dotted), fitted constants can be found in Table 2. Red and blue circles respectively correspond to experimental observations from Mole et al. (1983) and Olme et al. (2013b). Note that the rAML onset times following 1 Gy and 2.5 Gy exposure were based on pooled observations over the range of 0.25–1.50 Gy and 2–3 Gy irradiated CBA/H mice correspondingly.

Predicted cumulative rAML incidence following 2.5 Gy (blue) and 4.5 Gy (red) exposure is in line with experimental observations of X-ray irradiated CBA/H mice following 2–3 Gy (dashed blue stairs) and 4.5 Gy (dashed red stairs) exposure (Mole et al. 1983, Fig. 4c). Figure 4d shows that dose- and time-dependent cumulative rAML model predictions can be adequately approximated by a normal CDF with the aforementioned functions for the mean ($\mu _{t_{AML}}(D)$) and the SD ($\sigma _{t_{AML}}(D)$). The 2.5th, 25th, 50th, 75th and 95th rAML onset time percentile contours are shown as a function of dose and time for the normal CDF (solid curve) and the actual model predictions (dotted). The shown percentiles decrease in a nonlinear fashion with dose.

### The linear-quadratic rAML dose-response curve

The dose-response for murine rAML was calculated by scoring all the cases observed after running the model 100 million times for each dose up to 0.2 Gy and 10 million times for any other dose of interest (black curve, Fig. 5a). Model results are in line with data used in the fitting procedure derived from four independent experiments (filled circles) in which male CBA/H mice were exposed to various X-ray doses (Major and Mole 1978; Mole et al. 1983). Open circles represent data points from Mole et al. (1983) not included in the fit due to the absence of replicates. Peak rAML incidence of about 22% was observed following 2.6 Gy exposure, which is similar to the dose required to reach maximum del2 formation (Fig. 3b). Further increasing the absorbed dose decreased rAML onset due to depletion of del2 cells and competing causes of death. Note that the relatively high rAML incidence following 3 Gy exposure is directly responsible for the slight underestimation of CBA/H mouse survival time post-irradiation shown in Fig. 4a. The purple, red and blue curves respectively correspond to the rAML incidence diagnosed 50, 75 and 100 weeks after exposure, revealing that approximately 50% of the maximum incidence is diagnosed around 75 weeks post-IR, which is expected based on the median time of rAML onset shown in Fig. 4d.

Figure 5b shows that the LD rAML model solution (open circles) can be accurately described using an LQ model (red, $\alpha =3.63$ Gy^-1^, $\beta =10.1$ Gy^-2^) with parameters derived from a fitting procedure with logarithmic transformed LD incidence estimates, yielding $RSS_{log}=0.0001$. It was additionally assessed how well this LQ LD response curve can be reproduced when only HD rAML model solutions (black-filled circles) between 0.25 and 1.00 Gy were made available in the fitting procedure to extrapolate from. Slight overestimation of LD rAML was then found through the LQ model (blue, $\alpha =4.70$ Gy^-1^, $\beta$ = 6.51 Gy^-2^, $RSS_{log}=0.53$). The LD rAML model solution can be described more accurately when rAML is assumed to be proportional to the mean number of viable del2 cells *I* present per mouse after IR exposure (purple, Eq. 8), yielding $RSS_{log} = 0.10$ for a proportionality constant of 0.107. Inferior results were found when trying to reconstruct LD rAML incidence through HD rAML model solutions without application of a logarithmic transformation in the curve fitting procedure, leading to $RSS_{log}$ values of 1.57 and 0.23 for the LQ model ($\alpha = 5.44$ Gy^-1^, $\beta = 5.48$ Gy^-2^) and the del2 induction function respectively.

As expected, overestimation of the LD rAML model solution occurs when inferring possible incidence with linear models acquired through a fitting procedure with ($\alpha _L=8.61$ Gy^-1^; $RSS_{log}=8.61$) and without ($\alpha _L=9.81$ Gy^-1^; $RSS_{log}=12.4$) log transforming the HD rAML model solution. These results could subsequently be improved by dividing the LD extrapolations, made with the linear models, by the LDEF estimated in 1 Gy, producing $RSS_{log}$ values of 1.61 (LDEF= 2.39) and 0.44 (LDEF = 2.01) respectively. In the case of a log transformation, the LDEF of 2.39 produces a transformed slope parameter of $\alpha _L/LDEF=3.60$ Gy ^-1^, note that this is approximately equal to the aforementioned true LD slope parameter $\alpha =3.63$ Gy^-1^. Obviously, a linear model lacks a quadratic component with a beta-coefficient, resulting in an increasingly severe underestimation of true rAML incidence when increasing the dose from 0 to 0.20 Gy. Hence explaining the relatively large $RSS_{log}$ value of 1.61. Without log transforming the HD rAML model solution, the LDEF of 2.01 yields a transformed linear slope parameter of $\alpha _L/LDEF=4.88$ Gy^-1^. This is an overestimation of the true LD slope parameter $\alpha$, resulting in a relatively small $RSS_{log}$ value of 0.44 because the quadratic component of the true dose-response curve is now only slightly underestimated for doses between 0.14–0.20 Gy (results not shown).

## Discussion

The mathematical two-hit model of murine leukemogenesis presented here is a more realistic extension of a previous modeling effort by Dekkers et al. (2011). The model was extended by explicitly including cell survival and del2 induction in terms of the LQ model and by coupling bone marrow leukemogenesis to a survival model in which mice can die from other causes than rAML. Obtained results on cell survival, del2 formation, survival time and rAML incidence/onset are in good agreement with experimental observations (Mole et al. 1983; Major 1979; Olme et al. 2013a; Mohrin et al. 2010). Note, however, that the presented model is mostly based on historical CBA/H mouse data (Major 1979; Mole et al. 1983) because of the availability of dose-dependent rAML incidence and survival time data. This resulted in relatively high rAML incidence and late rAML onset times when compared to recent experiments also conducted with CBA/H mice and the same radiation quality (Olme et al. 2013a; Verbiest et al. 2018b). This disparity could possibly be attributed to differences in housing conditions, rAML diagnosis protocol and (humane) endpoints for sacrificing laboratory animals.

Even though the model can be used to reproduce experimental observations this does not necessarily signify that the underlying model assumptions are correct, since it remains a simplified representation of murine rAML, as illustrated by the following three examples. Firstly, similarly to Dekkers et al. (2011), it was assumed here that HSCs are the target cells at risk for bringing about rAML. However, it is possible that the actual target cells are from a different HSPC subpopulation. If this is indeed the case, then the model can still be applied to murine rAML by adjusting the initial number of healthy target cells *H* and repeating the parameter fit analysis. The survival curve for HSCs is quite similar to HSPCs (Mohrin et al. 2010), indicating that an adjustment of LQ survival parameters might not be necessary. Secondly, it was assumed that $\textit{in vitro}$ HSC survival, described through the LQ model, is a good predictor for $\textit{in vivo}$ loss of clonogenic potential. As a consequence it was automatically assumed that cell survival under normoxic conditions ($pO_2\approx$140 mmHg) is similar to bone marrow conditions ($pO_2\approx$22 mmHg, Spencer et al. 2014). This is supported by the observation that cellular radiosensitivity remains relatively unchanged as a result of such a difference in oxygen tension (Stewart et al. 2011; Hall and Giaccia 2011). Finally, Yates et al. (2017) discussed that Gillespie’s algorithm is often used improperly to describe processes, such as cell proliferation, for which the inter-event time does not follow an exponential distribution. Relatively short generation times can then be sampled from the exponential distribution, resulting in overestimation of the expected number of cells compared to what one should observe based on an exponential growth model. It is noted that in the present study an adaptive tau-leap algorithm developed by Cao et al. (2006) was used that only switches to Gillespie’s algorithm when few cells are present. To test whether low cell counts associated with LD exposure affected the rAML incidence, the switch to Gillespie’s algorithm was disabled such that there was no deviation from the expected exponential growth curve. From this it is concluded that one could use the much faster adaptive tau-leap algorithm because the results were identical in both implementations.

Not only was it possible to confirm previously made observations indicating that the latency between irradiation and observable rAML decreases with the absorbed dose (Major 1979; Upton et al. 1958), but also to describe dose-dependent rAML onset time through a simple normal CDF. In the model used here, the mean time to rAML onset initially reduced with dose due to a large increase in the total number of cells harboring the interstitial deletion with Sfpi1 loss; therefore increasing the pool of pre-leukemic cells that have the potential to become leukemogenic. Although further increasing the dose resulted in a decrease of the number of viable del2 cells, this did not translate to higher average rAML onset times. Instead, the mean time to rAML diagnosis kept on decreasing because the murine survival time decreased with dose and, as a result, the mice that were diagnosed with rAML had to develop the malignant cell transformation early on.

The numerical solutions of the model presented here were the result of time consuming simulations during which millions of *in silico* mice were irradiated. Numerical solutions were approximated with simple functions to allow for easy comparison and to study the possible form of the dose response curve. An LQ function was found to accurately describe the LD rAML response curve calculated with the CBA/H male mouse model. It was further tested how well functions can accurately reconstruct LD model incidence when only the HD model solution was made available to extrapolate from. An LQ model and a function for describing rAML in terms of the number of IR-induced del2 cells per mouse were considered, both were able to quite accurately reproduce LD rAML incidence with the latter slightly outperforming the former. Suggesting that the first hit in the two-hit model of rAML is largely responsible for determining the form of the LD response curve. Verbiest et al. (2015) discussed the differences between human primary AML and murine rAML, describing that heterozygous mutations in the human analogue of murine Sfpi1 are rare (Mueller et al. 2002; Bonadies et al. 2010). The dose-dependent expression for IR-induced del2 formation function should therefore not be applied to human rAML.

Although epidemiological studies have shown that the rAML dose-response curve is probably nonlinear (Preston et al. 1994; Richardson et al. 2009; Hsu et al. 2013), the assumption of linearity remains practical for the purpose of radiation protection (Boice 2017). The LDEF allows one to account for the possible overestimation of risk when using a linear model for a dose-response curve that might actually be linear-quadratic in radiation dose (SSK 2014). Here, the LDEF was applied to a linear dose-response line fitted to model-derived HD incidence estimates between 0.25 and 1 Gy to perform a simple examination of how well this factor facilitates LD extrapolation. In general, the LD rAML incidence curve derived from the presented mathematical model could be better approximated when model parameters were derived from a fitting procedure with log transformed incidence estimates. This improvement was obtained because a logarithmic transformation reduces the weight of larger incidence values in a curve fitting procedure. As one might expect, a linear model is not suitable for describing model-derived LD rAML incidence. The actual LD response curve could be described more accurately with a linear model after dividing the LD extrapolations with the LDEF, estimated at 1 Gy. Although an improvement was obtained, utilization of the LDEF additionally resulted in the (slight) underestimation of model-derived rAML incidence over a specific dose range. The dose range over which one might underestimate/overestimate true rAML incidence is dependent on used data transformations *and* the chosen reference dose for estimating the LDEF. Although an LQ model is more difficult to use in radiation protection compared to a linear model with an LDEF. The LQ model should ideally be used when one is interested in describing LD incidence when only HD data are available to extrapolate from *and* when there are indications that the LD curve might be LQ in radiation dose. It should be noted that the presented LDEF analysis is limited due to the use of noise-free model-derived rAML estimates.

Hsu et al. (2013) conducted a comprehensive epidemiological study on the mortality from various forms of leukemia among Japanese atomic bomb survivors dependent on factors such as dose, age at exposure and sex. A purely quadratic function was used as the preferred model to describe excess absolute/relative rAML risk over a wide dose-range. A similar finding for excess relative rAML risk was found by Richardson et al. (2009), revealing that the addition of a linear term to a purely quadratic dose-response function contributed little to the model fit. The model presented here is based on the known murine two-hit rAML pathway in which only male CBA/H mice of the same age were exposed. The modeling effort described in the present paper can complement epidemiological studies by translating the (limited) understanding of leukemogenesis into a simplified mathematical model to subsequently study the possible dose-response curve. Although the present two-hit model results are in good agreement with the data, the obtained LQ dose-response curve follows from the made assumptions, and thus, might be incorrect. For example, a quadratic dose-response curve can be obtained with the model if the Sfpi1 point mutation rate is made proportional to dose without an offset, making the occurrence of rAML following LD exposure rare due to a small dose-dependent mutation rate. An LQ curve is again obtained when the offset is not exactly zero. Thus, the dose-response curve found here should therefore be interpreted in light of the model assumptions that had to be made due to the many unknown factors possibly affecting murine rAML.

Murine rAML can largely be explained by the two-hit model of leukemogenesis, which is supported by the finding from Metcalf et al. (2006) that approximately 95% of C57BL mice surviving conditional-knockout of both Sfpi1 alleles developed AML. Furthermore, Finnon et al. (2012) found that about 77% of the rAML cases (n=30) in CBA/H and CBA/H x C57BL mice tested positive for del2. Of these mice, approximately 83% carried the R235 point mutation. Flt3-ITD mutations without Sfpi1 involvement were found in 10% of the rAML cases, indicating the existence of another possible murine rAML pathway. Recently, O’Brien et al. (2020) analyzed 123 murine rAML samples and revealed new rAML pathways involving Kras mutations and Sfpi1 promoter methylation. It was further confirmed that the Sfpi1 deletion followed by an R235 point mutation is the most common pathway (64% of rAML cases). Interestingly, about 4% of the rAML cases showed the Sfpi1 R235 mutation without a detectable deletion. The dose-response curve obtained here assumes that all rAML cases result from the major pathway of leukemogenesis. However, different disease pathways will probably have distinct dose response curves, with the overall form being a weighted average, which might differ across gender (Verbiest et al. 2018a; O’Brien et al. 2020). Further research is required to investigate the possibility of including multiple pathways.

The model presented here is a step towards quantifying possible murine rAML incidence dependent on dose-rate and various dose fractionation schedules. By incorporating these modes of IR exposure one can calculate dose- and dose-rate-dependent effectiveness functions relevant as a possible tool for predicting LD (rate) effects given the availability of only HD (rate) data. These functions could then possibly be used within the context of murine rAML to shed more light on the ongoing discussion surrounding the usage of reduction factors such as the LDEF, DREF and DDREF.

## Data Availability

The data used in the fitting procedure are available from the corresponding author upon request.
